# Opinions of the Press

**Published:** 1874-12

**Authors:** 


### OPINIONS OF THE PRESS.

We cannot publish all the good things said of us by the newspapers, but we must make room for the following appreciative remarks from the *Times*, Watseka, Ill.

Hall's Journal of Health for November comes crowded with good advice. The article on dyspepsia is sound, the cure sensible. The article on drinks would open one's eyes, especially so when the proportion of alcohol in the different drinks and bitters is given. Lowest on the list stands Schenck's Beer, which has 2 per cent. alcohol. Lager Beer has 4, New Cider 5, Vinegar Bitters 8, Temperance Bitters 18, California Bitters 19, Plantation Bitters 30, Hostetter's 43; Whisky has but 40 per cent. We wish we could reprint the entire book for our readers. We would not give it for any other exchange we have. The articles are all in plain, clear language, smoothly running, and so interesting and instructing, that when through reading one of them you immediately begin on the next. The article on food we will print in our next issue. According to an estimate made therein, skim cheese is the most nutritious food. The magazine, which comes monthly, and averages about thirty pages, costs but $2.00 per year. Published by E. H. Gibbs & Co., New York."

Also, the *Republican*, Kasson, Minn., tells its readers:

"Two dollars cannot be better invested by any family than by subscribing for this popular health magazine."

From the *Reporter*, Franklin Grove, Ill., thus:

"Hall's Journal of Health for November is on our table. We have files of this valuable health journal as far back as Vol. I. It is now in its twenty-first year., The longer we read it the better we like it. Unlike many kindred publications, it has no "ism" or "ology" to advocate. Its rules of health are based on common sense principles. Terms, $2 year."

